# Unveiling Versatile Applications and Toxicity Considerations of Graphitic Carbon Nitride

**DOI:** 10.3390/ijms25147634

**Published:** 2024-07-11

**Authors:** Alexandra Paulína Drdanová, Timea Ema Krajčovičová, Miroslav Gál, Katarína Nemčeková, Zuzana Imreová, Jozef Ryba, Monika Naumowicz, Tomáš Homola, Tomáš Mackuľak, Veronika Svitková

**Affiliations:** 1 Department of Environmental Engineering, Faculty of Chemical and Food Technology, Slovak University of Technology in Bratislava, 812 37 Bratislava, Slovakia; alexandra.drdanova@stuba.sk (A.P.D.); zuzana.imreova@stuba.sk (Z.I.); tomas.homola@stuba.sk (T.H.); tomas.mackulak@stuba.sk (T.M.); 2 Department of Inorganic Technology, Faculty of Chemical and Food Technology, Slovak University of Technology in Bratislava, 812 37 Bratislava, Slovakia; timea.krajcovicova@stuba.sk (T.E.K.); katarina.nemcekova@stuba.sk (K.N.); veronika.svitkova@stuba.sk (V.S.); 3 MicroPoll s.r.o., 812 43 Bratislava, Slovakia; jozef.ryba@stuba.sk; 4 Department of Polymer Processing, Faculty of Chemical and Food Technology, Slovak University of Technology in Bratislava, 812 37 Bratislava, Slovakia; 5 Department of Physical Chemistry, Faculty of Chemistry, University of Bialystok, 15-245 Bialystok, Poland; monikan@uwb.edu.pl

**Keywords:** g-C_3_N_4_, nanosheets, synthesis, modification, application, toxicity

## Abstract

Metal-free, low-cost, organic photocatalytic graphitic carbon nitride (g-C_3_N_4_) has become a promising and impressive material in numerous scientific fields due to its unique physical and chemical properties. As a semiconductor with a suitable band gap of ~2.7 eV, g-C_3_N_4_ is an active photocatalytic material even after irradiation with visible light. However, information regarding the toxicity of g-C_3_N_4_ is not extensively documented and there is not a comprehensive understanding of its potential adverse effects on human health or the environment. In this context, the term “toxicity” can be perceived in both a positive and a negative light, depending on whether it serves as a benefit or poses a potential risk. This review shows the applications of g-C_3_N_4_ in sensorics, electrochemistry, photocatalysis, and biomedical approaches while pointing out the potential risks of its toxicity, especially in human and environmental health. Finally, the future perspective of g-C_3_N_4_ research is addressed, highlighting the need for a comprehensive understanding of the toxicity of this material to provide safe and effective applications in various fields.

## 1. Introduction

Graphitic carbon nitride (g-C_3_N_4_), as the most stable and resistant allotrope out of five polymorphs of carbon nitrides (α-C_3_N_4_, β-C_3_N_4_, cubic-C_3_N_4_, pseudocubic-C_3_N_4_, and graphitic-C_3_N_4_), is a novel metal-free organic semiconductor with several notable physiochemical properties. In the last decade, g-C_3_N_4_ has attracted remarkable attention in research due to its prospective application in numerous fields such as photocatalysis, energy storage, electrochemical sensors, and biomedical engineering. The attribute “graphitic” refers to the layered structure with individual heterocyclic layers bound together by weak van der Waals forces like graphite. The hexagonal network consisting of C-N covalent bonds is mainly formed by s-triazine (triazine) or tri-s-triazine (heptazine) units. The general formula C_3_N_4_ could be misleading, as the theoretical C/N ratio of 0.75 is hardly obtained by synthesis and the g-C_3_N_4_ also contains a considerable amount of hydrogen from amino uncondensed groups. With the convenient band gap energy of ~2.7 eV, g-C_3_N_4_ is an attractive photocatalyst active under the irradiation of visible light, suitable for systems utilising sunlight [1,2,3,4].

To date, many reviews have already been published on the topic of g-C_3_N_4_, covering synthesis methods, strategies for performance improvement, and applications in various fields [5,6,7,8]. There are considerably fewer reviews summarising the current knowledge and research concerning the toxicity and biocompatibility of this material. In this review, we first describe the fundamental methods of synthesis of both bulk and exfoliated g-C_3_N_4_ nanomaterials. Subsequently, we aim to provide a brief overview of the latest research on diverse applications of g-C_3_N_4_, mainly on electrochemical and photocatalytic domains. Lastly, we focus on the summary of the toxicity of this material, discovered so far through various toxicity assays, namely, in vitro toxicity assessment on human and animal cells and ecotoxicity tests on animals, microorganisms, and plants.

## 2. Preparation Methods of g-C_3_N_4_

**Figure 1:**
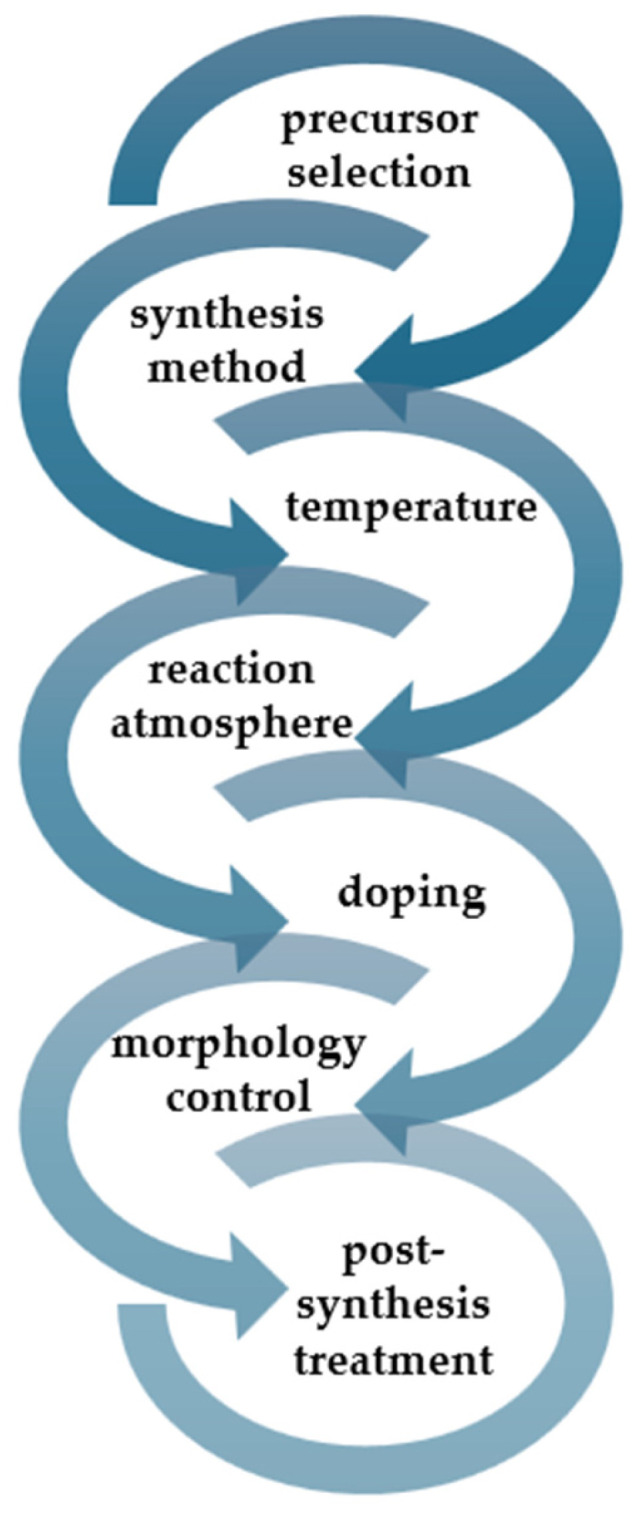
The key synthesis factors influencing the final properties of g-C_3_N_4_.

Evaluating the resource efficiency of g-C_3_N_4_ synthesis methods is essential for assessing its overall sustainability. Green synthesis routes that minimise energy consumption, waste generation, and the use of hazardous materials are preferred. The synthesis of g-C_3_N_4_ is influenced by several key factors that determine its structural, morphological, and functional properties. These factors include the choice of precursors, synthesis method, temperature, and reaction atmosphere (Figure 1). By carefully controlling these factors, researchers can tailor the properties of g-C_3_N_4_ to suit specific applications, ranging from photocatalysis and water splitting to sensors and biomedical applications.

### 2.1. General Conditions and Precursors of g-C_3_N_4_ Green Synthesis

One of the most commonly used methods of g-C_3_N_4_ synthesis is the calcination of various nitrogen- and carbon-rich precursors, generally melamine, dicyandiamide, thiourea, and urea, under the O_2_ or N_2_ atmosphere [4,9]. These precursors undergo thermal polymerisation or condensation reactions, occurring between 400 °C and 600 °C, while the building units and g-C_3_N_4_ polymer network fully form above 500 °C. When passing the temperature of 600 °C, the polymer becomes unstable, and beyond 700 °C it is decomposed completely [10].

During the preparation of g-C_3_N_4_, the most important factors that influence the composition and properties of the end product are precursor selection and polymerisation conditions, i.e., the temperature of calcination and heating rate. Zheng et al. conducted a study in which melamine and urea were chosen and compared as reagents for g-C_3_N_4_ formation solely through thermal polymerisation at varying temperatures (500, 550, 600, and 650 °C). It was found that the surface area of g-C_3_N_4_ gradually increased with an increasing pyrolysis temperature in both cases; however, urea-derived g-C_3_N_4_ was overall a lot less dense and lighter, with a highly porous, “sponge-like” structure in comparison to melamine-derived g-C_3_N_4_ with a high order of crystallinity. This phenomenon is attributed to a much larger atmosphere of gases (NH_3_, H_2_O, CO_2_) generated from the pyrolysis of urea, creating bubbles that may function as gas templates, which increases the distance between the layers. Samples derived from urea also showed better adsorption and photocatalytic abilities [11].

More than the calcination temperature itself, a major factor in controlling the outcome of g-C_3_N_4_ products may be the heating rate during polymerisation. Sharma et al. compared g-C_3_N_4_ prepared from urea and melamine at varying heating rates. They found that g-C_3_N_4_ synthesised from both urea and melamine showed enhanced photocatalytic and surface properties when produced at a slower heating rate of 2 °C/min in comparison with g-C_3_N_4_ prepared at a high heating rate of 10 °C/min [12]. In addition to the calcination described above, i.e., the thermal polymerisation of nitrogen- and carbon-rich precursors, other methods for the preparation of g-C_3_N_4_ are known, including solvothermal and hydrothermal methods, molten salt synthesis, and chemical vapour deposition (CVD). In solvothermal synthesis, g-C_3_N_4_ is prepared in a solvent (either water or an organic solvent) under high pressure and temperature, which can result in various morphologies of g-C_3_N_4_, such as nanosheets or g-C_3_N_4_ with a porous structure [13]. Using the molten salt method, g-C_3_N_4_ with a high degree of crystallinity and a large surface area can be prepared. In this technique, molten salts at high temperatures, such as chlorides, nitrates, and carbonates, are utilised as the reaction medium. This method is also often used in the preparation of g-C_3_N_4_ nanosheets [14]. CVD can be utilised to form a thin film of g-C_3_N_4_ on a suitable substrate using gas-phase precursors at elevated temperatures. This method yields thin g-C_3_N_4_ films with controlled thickness and excellent uniformity [15].

### 2.2. Preparation of g-C_3_N_4_ Nanomaterials

Bulk g-C_3_N_4_ without further treatment, activation, or modification exhibits inadequate photocatalytic properties due to a relatively low surface area and few active sites, low solar-light adsorption, and fast recombination of electron–hole pairs [16]. There is an important emphasis on the preparations of g-C_3_N_4_ nanomaterials mainly in the form of nanosheets or even nanotubes, which exhibit noticeably enhanced physiochemical properties compared to bulk g-C_3_N_4_. Furthermore, for improved properties of g-C_3_N_4_, elemental doping with nonmetals (B, S, O) or metals (Ni, Co, Mg) or doping with various molecules and materials (TiO_2_, carbon nanotubes) to introduce certain functional groups into the structure of g-C_3_N_4_ and many other methods are also being explored as means of g-C_3_N_4_ functionalisation for technological applications [17,18,19].

**Figure 2:**
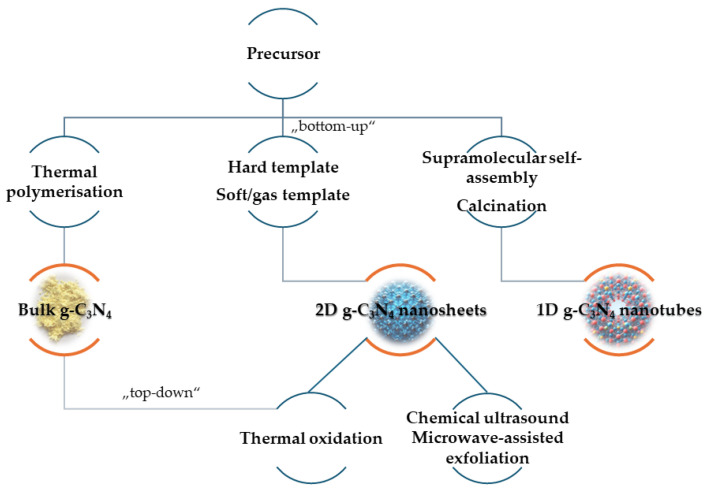
Schematic illustration of the synthesis process of g-C_3_N_4_ resulting in different types of nanomaterial.

Nanosheets of g-C_3_N_4_ with a hexagonal lattice similar to graphene are typical examples of 2D nanomaterials, and their formation can be achieved through two different approaches, regarded as the “top-down” and “bottom-up” methods (Figure 2). When utilising a “top-down” method, the layered bulk material is delaminated into nanosheets. Nanosheets of g-C_3_N_4_ with a thickness of around 2 nm can be obtained by simple thermal oxidation of bulk g-C_3_N_4_ in the air atmosphere [20]. The other most commonly used top-down methods are chemical, ultrasound, or microwave-assisted exfoliation [21,22]. In contrast, a “bottom-up” method typically relies on the use of various templates to build the desired materials out of the most elementary units—atoms and molecules. In the hard-template method, a rigid template of pre-synthesised material, e.g., silica, alumina, or clays, acts as a mould to give shape to the precursor material. On the other hand, soft templating relies on the use of surfactants, block copolymers, and ionic liquids to act as templating agents to assist in the assembly of the precursor material into a porous structure [23]. Gas-template methods could also be included in bottom-up soft-template methods. Easy one-step synthesis with the use of NH_4_Cl as a gas-template agent could be used for the preparation of 3.1 nm g-C_3_N_4_ nanosheets directly from dicyandiamide as a precursor [24]. The main advantages of the top-down approach are scalability and reproducibility, while the bottom-up approach enables the direct synthesis of nanosheets with uniform size and crystallinity [25]. In addition to 2D g-C_3_N_4_ nanosheets, 1D nanotubes of g-C_3_N_4_ have also shown significant performance enhancements. Mo et al. successfully prepared nitrogen-rich g-C_3_N_4_ with a tubular structure by calcination of a supramolecular precursor under an NH_3_ atmosphere. The precursor for g-C_3_N_4_ nanotubes was prepared using the hydrothermal method through the supramolecular self-assembly of melamine with hydroxylammonium chloride. The resulting g-C_3_N_4_ nanotubes exhibited a significantly increased specific surface area, approximately 16 times higher than bulk g-C_3_N_4_ [26].

As has been shown, the synthesis of g-C_3_N_4_ can be achieved through various methodologies, each affecting the material’s efficiency, morphology, and purity. Each synthesis method offers distinct advantages and trade-offs, impacting the suitability of g-C_3_N_4_ for specific applications such as photocatalysis, water splitting, and sensor technologies. These are summarised in Table 1.

## 3. Applications of g-C_3_N_4_

**Figure 3:**
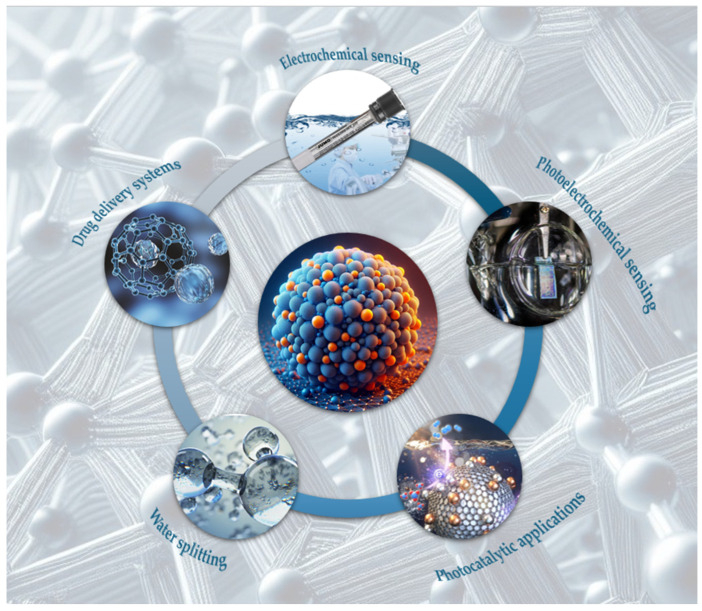
Schematic illustration of g-C_3_N_4_ applications.

g-C_3_N_4_ showcases outstanding multifunctionality and enhanced performance in various applications, positioning it as a leading material in photocatalysis and related fields (Figure 3).

In electrochemical sensing, g-C_3_N_4_-modified electrodes exhibit high sensitivity and selectivity for detecting organic compounds and heavy metals. The enhanced detection limits are attributed to its high surface area and conductivity, making g-C_3_N_4_-modified electrodes more effective than traditional ones. This performance improvement ensures faster, cheaper, and more reliable in situ analysis, critical for applications in environmental monitoring and healthcare diagnostics.

Photoelectrochemical (PEC) sensing benefits from g-C_3_N_4_’s role as a photoactive electrode. It enhances PEC system efficiency by generating effective photogenerated electron–hole pairs. This results in higher sensitivity and specificity compared to many conventional materials. g-C_3_N_4_’s ability to improve PEC sensing performance is particularly advantageous in detecting low concentrations of analytes, making it suitable for applications in food safety testing and pollution monitoring.

In photocatalytic applications, g-C_3_N_4_ is effective in pollutant degradation and water disinfection, offering an eco-friendly alternative to traditional methods. While bulk g-C_3_N_4_ has limitations, its nanostructured forms exhibit enhanced photocatalytic activity, often surpassing other photocatalysts in efficiency. This makes g-C_3_N_4_ an excellent choice for environmental remediation, where sustainable and efficient solutions are needed.

For water splitting, g-C_3_N_4_ nanocomposites demonstrate high efficiency in hydrogen production. Their tunable bandgap and stability make them competitive with other photocatalytic materials. The ability to efficiently split water into hydrogen and oxygen positions g-C_3_N_4_ as a key material in the development of the hydrogen economy, which is crucial for addressing climate change and reducing reliance on fossil fuels.

In drug delivery systems, g-C_3_N_4_ nanoparticles are valued for their biocompatibility and functionalisation capability. These properties enable targeted drug delivery with controlled release, offering improved therapeutic efficacy over some traditional drug delivery systems. The use of g-C_3_N_4_ in biomedical applications highlights its potential to enhance the effectiveness and precision of treatments.

Phototherapy is another area where g-C_3_N_4_ excels due to its superior light absorption and photothermal conversion efficiency. This material provides a non-invasive treatment option with potential advantages over conventional photothermal agents, particularly in cancer treatment and other medical applications. g-C_3_N_4_’s properties ensure effective and safe phototherapy, promoting better patient outcomes.

### 3.1. Electrochemical Sensing

The basic principle of any electrochemical sensor or biosensor is the ability to convert chemical signals into easy-to-interpret electrical signals, therefore offering a fast, cheap, and selective in situ analysis. To reach even lower limits of detection, elevated sensitivity, and selectivity, extensive research is being put into the development of modified commercially available electrodes. Properties such as high specific surface area and conductivity, facile surface functionalisation, tunable bandgap, and exceptional thermal and chemical stability are only some that are typically highlighted within the topic of g-C_3_N_4_ as an electrode modifier. Several formats of electrodes with g-C_3_N_4_ modifications have recently been prepared for the detection of a plethora of substances [28].

A carbon paste electrode (CPE) consisting of graphite and paraffin wax, along with g-C_3_N_4_ as an attractive modifier for the heightened signal response, was used for the detection of tryptophan [29] and acyclovir [30]. Highly accurate determination of heavy metals (Cr, Ni) in tap water was accomplished by potentiometric titration with g-C_3_N_4_ and multiwalled carbon nanotube (MWCNT)-modified CPE [31].

Screen-printed electrodes (SPE) modified solely with nanoscale g-C_3_N_4_ doped with additional oxygen-containing functional groups were utilised for dibenzofuran analysis via impedimetric detention to achieve an exceptionally low LOD (pM range) [32]. In another study, Cu, Zn, and Fe metal–organic frameworks (MOFs) enriched with melamine-derived g-C_3_N_4_ were evaluated towards 2,4-dichlorophenol sensing on a modified SPE. The Fe-MOF/g-C_3_N_4_ nanocomposite showed the most fitting electrochemical behaviour and was used for real sample analysis, achieving nanomolar detection limits [33]. Niaz et al. used cathodic stripping voltammetry for the detection of iodide ions (I^−^) on a chitosan/g-C_3_N_4_ composite-modified SPE. After applying a cleaning potential, the modifying layer remained unchanged for several repeated measurements, which stands out among many single-use sensing methods utilising SPEs [34].

Glassy carbon electrodes (GCE) are arguably the most widely used electrodes in electroanalytical sensing, with endless potential for surface modification to suit the specific choice of analyte. Metronidazole was successfully detected with appropriate selectivity with no interference from other electroactive compounds, using a MoS_2_/g-C_3_N_4_ composite anchored with Nafion on the electrode’s surface [35]. A GCE modified with a nanocomposite of Bi_2_Te_3_/g-C_3_N_4_ achieved some of the best limits of detection (calculated as 0.7 μM) reported to date for the electrochemical analysis of epinephrine, also known as adrenaline [36]. Impressive results for perphenazine determination were also achieved via a LaCoO_3_/g-C_3_N_4_-modified GCE. In this work, the nanocomposite resembled “belts” of individual sheet-like layers of g-C_3_N_4_ dotted with circular nanoparticles of the lanthanum compound [37].

Each electrochemical sensing application has unique requirements and challenges. Optimising g-C_3_N_4_-modified electrodes for specific analytes, matrix compositions, and environmental conditions remains a challenge. Tailoring the properties of g-C_3_N_4_ to address these specific application needs requires further investigation. One of the primary challenges associated with g-C_3_N_4_-modified electrodes is the complexity and variability of synthesis methods. Achieving consistent and reproducible g-C_3_N_4_ structures with desired properties can be challenging, impacting the reliability and reproducibility of electrode performance. While g-C_3_N_4_ shows promise for electrochemical sensing applications in research settings, scaling up production and commercialisation remain hurdles. Developing cost-effective synthesis methods and ensuring scalability while maintaining performance is crucial for transitioning g-C_3_N_4_-modified electrodes from the lab to practical applications.

### 3.2. Photoelectrochemical Sensing

Photoelectrochemical (PEC) detection utilises light for excitation and measures current or voltage as the output signal. In the construction of PEC platforms, g-C_3_N_4_ can have different roles, such as a photoactive electrode material, sensitiser, and either electron donor or acceptor. Additionally, owing to diverse surface functional groups, g-C_3_N_4_ can act as an immobiliser for bioreceptors such as aptamers [38,39].

A PEC system was developed on an F-doped SnO_2_ (FTO) glass platform first modified with g-C_3_N_4_ and BiOI on top for accurate detection of ascorbic acid. The continuously decreasing cathodic photocurrent was attributed to an increasing ascorbic acid concentration. The research states that this combination of electrode modifiers leads to the formation of abundant charge transfer pathways. Their reordering or subsequent layering creates a template for task-specific modulation of these pathways based on the intended use [40].

Surface-modified g-C_3_N_4_ nanosheets decorated with clusters of Cu (2%) provided an accurate photoelectrochemical sensing platform for the detection of 4-nitrotoluene. Cu (II) ions effectively worked in synergy with the exfoliated g-C_3_N_4_ nanosheets to promote separation and migration of the photogenerated electron–hole pairs, therefore greatly enhancing the photocurrent response to the analyte. The copper ions in this system served as an electron-trapping medium to facilitate the reduction of p-nitrotoluene. The surface modification proved to be crucial in this system, as g-C_3_N_4_ nanosheets themselves showed minimal change in photocurrent in response to increasing 4-nitrotoluene concentration [41].

A photoelectrochemical aptasensing platform for the detection of extremely low concentrations of atrazine was developed by Yan et al. In this study, additional cyano functional groups, as well as nitrogen deficiencies, were introduced into the bulk g-C_3_N_4_ material to trap photoinduced electrons, improve the adsorption capacity of visible light, and therefore improve PEC performance. Atrazine-specific aptamer as the identification element was further used to modify g-C_3_N_4_, and atrazine concentration was relative to the decrease in detected photocurrent [42]. Tian et al. prepared an aptasensor based on the electrochemiluminescence (ECL) of g-C_3_N_4_ for the accurate detection of aflatoxin B1. The biosensor was formed by deposition of g-C_3_N_4_ modified with aflatoxin-specific aptamer on GCE. By itself, g-C_3_N_4_ exhibited intense ECL signals; therefore, growing aflatoxin concentration was directly proportional to a decrease in ECL signal response, as the aflatoxin–aptamer complex blocked active sites on the material [43].

Achieving precise control over surface functionalisation and maintaining the stability of modified g-C_3_N_4_ structures are significant challenges that warrant attention in PEC sensing applications. Potential interference from other compounds and optimised selectivity for real-world applications are critical considerations for the practical implementation of g-C_3_N_4_-based PEC sensors. As with electrochemical sensing, scaling up the production and commercialisation of g-C_3_N_4_-modified PEC platforms presents challenges. Developing cost-effective synthesis methods and ensuring scalability while maintaining performance and reproducibility are essential for translating laboratory-scale research into practical sensing devices.

Table 2 summarises the aforementioned applications of several g-C_3_N_4_-based sensors including their comparison to methods using different modifiers on the electrode surface. In most cases, sensors utilising g-C_3_N_4_ were able to achieve higher sensitivity and significantly lower limits of detection to the same analyte. Additionally, in several examples, the modifier containing g-C_3_N_4_ requires fewer steps and simpler synthesis approaches, proving the practicality of this material in electrochemical and photoelectrochemical sensing.

### 3.3. Photocatalytic Applications

Among the first discovered and most prominently researched attributes of g-C_3_N_4_ is its photocatalytic properties; however, as has been mentioned, g-C_3_N_4_ in its bulk form is severely hindered for these applications, showing a relatively small specific surface area and therefore insufficient active sites as well as the rapid recombination of excited electron–hole pairs. Numerous studies over the years show that the limitations of bulk g-C_3_N_4_ can be easily worked around with the introduction of g-C_3_N_4_-based nanostructures or by engineering electron redistribution by introducing vacancies or doping g-C_3_N_4_ with other elements. It has been noted that many approaches to modifying g-C_3_N_4_ for enhanced photocatalytic properties rely on creating structures with various metals, which may be detrimental to the environment in the long run due to the low availability of some metals in nature and their inherent toxicity. Special attention is being paid to developing g-C_3_N_4_ catalysts using methods and materials with sustainability in mind [56,57].

A novelty approach to 1,2 amino alcohol synthesis has been developed through ultrathin porous g-C_3_N_4_ nanosheets-enabled photocatalysis under visible light by Xu et al. The 1,2-amino alcohol component serves as a crucial chemical backbone in numerous natural products, biologically active compounds, and ligands. This approach enables simultaneous reductive and oxidative reactions, leading to the formation of two radical intermediates that couple to produce the final product with almost 100% yields. Most notably, the synthesised photocatalyst exhibited satisfactory recyclability, remaining effective for up to seven cycles. Additionally, the reaction mechanism was explored for its applicability to other complex molecules, suggesting potential industrial applications for ultrathin porous g-C_3_N_4_ nanosheets in the synthesis of valuable pharmaceutical intermediates [58].

Ozonation refers to the production of reactive oxygen species (ROS) from ozone (O_3_) molecules, capable of attacking and degrading a wide range of organic compounds and microorganisms. Nanoclustered g-C_3_N_4_ functionalised with additional oxygen-containing (mainly C=O and C–O) groups was found to be an exceptional catalyst, capable of generating primarily ^•^OH and O_2_^•−^ radicals in the presence of O_3_, as confirmed by EPR analysis. When tested, atrazine removal of a system containing oxygen-functionalised g-C_3_N_4_ and O_3_ reached nearly 93% compared to O_3_ alone (63%). The material’s catalytic abilities remained constant under both acidic and alkaline conditions, suggesting a wide applicability [59]. Another study reports significantly lower (around 30%) usage of O_3_ and complete inactivation of *Escherichia coli* bacteria and JC virus in water, utilising a dual system of g-C_3_N_4_/O_3_, greatly reducing the cost of such water treatment. The main ROS generated were identified to be ^•^OH [60].

Xu et al. prepared g-C_3_N_4_ via a multiple-step calcination route resulting in a material with a combined crystalline and amorphous structure and a narrower bandgap than its bulk g-C_3_N_4_ counterpart, making it more conductive. Upon stimulation with xenon lamplight in an environment abundant in O_2_, g-C_3_N_4_ with a combined crystalline and amorphous structure was capable of efficient H_2_O_2_ production and nearly 100% removal of methylene blue under simulated conditions. The key ROS generated and important in the mechanism of pollutant degradation reaction were identified to be O_2_^•−^ radicals and singlet oxygen ^1^O_2_ [61].

Photocatalytic disinfection strategies against microbial and bacterial pollution have been considered recently as a preferable solution in contrast to traditional bacterial inactivation methods because of their ecological sustainability. Pure g-C_3_N_4_ without any additives shows certain antibacterial properties under visible light illumination on account of its unique photocatalytic properties. As previously mentioned, the photoexcited free radicals (^•^OH, O_2_^•−^) can attack cell walls and membranes, causing them to rupture, leak intracellular materials, and kill bacteria [62].

These approaches demonstrate innovative solutions to enhance the photocatalytic properties of g-C_3_N_4_, enabling its application in diverse environmental remediation and synthesis processes. However, concerns about the environmental impact of using metal-based modifications to enhance g-C_3_N_4_ photocatalytic properties are rising. It is important to develop sustainable g-C_3_N_4_ catalysts using methods and materials that minimise environmental harm. This reflects a growing awareness within the scientific community regarding the need for eco-friendly approaches to material synthesis and catalysis. Developing scalable and reproducible synthesis routes for g-C_3_N_4_-based nanostructures or doped materials is essential for practical applications but often presents challenges in terms of cost and complexity. Assessing the stability of these materials over extended operation periods and under different environmental conditions is crucial for evaluating their practical viability for industrial applications. Further research is needed to elucidate the underlying processes governing photocatalytic reactions on g-C_3_N_4_ surfaces and to identify key factors influencing catalyst activity and selectivity.

### 3.4. Watter Splitting

Another possible application of a photoelectrochemical system based on g-C_3_N_4_ is utilising the photogenerated electron–hole pairs to drive redox processes associated with water splitting. Simply put, water splitting is a chemical reaction in which hydrogen (H_2_) and oxygen (O_2_) are generated from water molecules. The development of economical and efficient means of water splitting is regarded to be one of the major building blocks for the hydrogen economy, considered to be one of the most promising ways to mitigate ongoing climate change caused by the use of fossil fuels [63]. Water splitting is divided into three main routes, photocatalytic, electrocatalytic, and photoelectrocatalytic. Photocatalytically, water splitting occurs through the formation of bound electron–hole pairs (excitrons) by adsorption of light. For electrocatalytic water splitting, O_2_ is generated on the anode and H_2_ is generated on the cathode at an electrode potential of 1.23 V, assuming the electrochemical cell has been assembled with appropriate materials. When these two principles combine, water splitting can also occur via a photoelectrocatalytic route, meaning a photocatalyst material is bound to an electrode and potential is applied to the system in the presence of light [64].

Wang et al. presented an interesting new approach to photoelectrocatalytic water splitting with the use of g-C_3_N_4_ nanofibers decorated with black phosphorous nanoparticles. Black phosphorous has the properties of a p-type semiconductor with notable optoelectrical qualities, with an adjustable bandgap ranging from 0.3 eV to 2 eV, depending on the specific morphology of the material, while g-C_3_N_4_ functions as a n-type semiconductor with a defined bandgap of 2.7 eV. The combination of these two materials as a p-n heterojunction led to the formation of a highly functional photoresponsive material, achieving a high evolution rate of hydrogen and being stable for over 8 h [65].

Sun et al. achieved exceptional H_2_ production rates through photocatalytically driven water-splitting reactions on a complex of g-C_3_N_4_ nanosheets deposited with single-atom cerium sites. In addition to the water splitting reaction, the prepared nanocomposite was able to oxidize amines after irradiation with visible light. The lifetime of photoinduced electrons was improved by 5 ns in comparison to bare g-C_3_N_4_, and the production of superoxide radicals was proven to be the driving force of the reaction mechanisms. The addition of cerium also improved charge transfer resistance and showed significant photoluminescence quenching compared to the bare g-C_3_N_4_ material [66].

The reaction of O_2_ evolution has been remarked on as highly energy-demanding ad not particularly fast due to its mechanism requiring the transfer of four electrons. Iridium oxide (IrO_2_) is the most commonly used catalyst in water electrolysis, but its main disadvantage is the low availability of the rare metal and, therefore, an exceptionally high price. Aiming to improve on these facts, Torres-Pinto et al. developed an electrocatalytic system consisting of g-C_3_N_4_ deposited onto Ni foam substrates. Upon extensive performance evaluation of this system, for the first time it was noted that a g-C_3_N_4_-based catalyst was able to surpass the point-of-reference catalyst IrO_2_ in terms of rates of generated O_2_ in an alkaline medium [67].

Table 3 summarises some advantages and disadvantages of g-C_3_N_4_ materials compared to other common catalysts. However, it should be noted that even though significant progress has already been made in this research, the use of g-C_3_N_4_ in photoelectrocatalysis is still in its beginning stages. Current trends focus on environmental friendliness and cost efficiency in technological materials suggesting great potential for g-C_3_N_4_-based catalysts.

### 3.5. Drug Delivery Systems and Phototherapy

Low biotoxicity and good biocompatibility make g-C_3_N_4_ a favourable material for biomedical applications, especially in the field of cancer treatment. It can be used as a nanocarrier by loading the therapeutic agent or drug into the cavities to make a complex. The drug molecules or therapeutic agents are loaded on the nanocarrier by weak forces of attraction, e.g., London dispersion force [72]. Assuming the acidic microenvironment of the tumour (a pH value around 5), both the anticancer drug and its carrier (g-C_3_N_4_) become negatively charged once absorbed by the tumour. Hence, the electrostatic repulsion facilitates the release of the anticancer drug. Recent insights into g-C_3_N_4_ in drug delivery testing have been summarised in more detail by Pourmadadi et al. [17].

Among many types of water-splitting materials, g-C_3_N_4_ has attracted considerable attention for its adjustable bandgap and band position. After the modification, water splitting can be driven under a high penetrable laser, which makes g-C_3_N_4_ suitable for phototherapy. Unfortunately, some disadvantages still restrict the application of g-C_3_N_4_ in water splitting, including a high recombination rate of photogenerated electron–hole pairs and lower light utilisation. Therefore, the modification design of g-C_3_N_4_ for further widening the light absorption range and improving stability is necessary to meet the requirements of practical water splitting [73].

## 4. Toxicity Considerations of g-C_3_N_4_

The mechanism of any phenol-like compound degradation using g-C_3_N_4_ typically involves a photocatalytic process wherein g-C_3_N_4_ serves as a catalyst to facilitate the decomposition of phenolic compounds under light irradiation [74]. When exposed to light, g-C_3_N_4_ absorbs photons, promoting electrons from the valence band to the conduction band, creating electron–hole pairs. The photoexcited electrons (e⁻) and holes (h⁺) can react with water and oxygen molecules adsorbed on the surface of g-C_3_N_4_ to generate already mentioned ROS. Phenol molecules present in the solution are adsorbed onto the surface of g-C_3_N_4_ through π–π interactions or hydrogen bonding. The ROS generated on the surface of g-C_3_N_4_ reacts with the adsorbed phenol molecules, leading to the oxidation of phenolic compounds into intermediate products such as hydroquinone, benzoquinone, and eventually, smaller organic acids, aldehydes, and carbon dioxide. During this process, several risks that can negatively impact the environment or human health need to be considered. These are summarised in Table 4.

To mitigate these risks, thorough research and risk assessments are necessary to understand the fate and toxicity of intermediate products, optimise photocatalytic conditions to minimise the formation of toxic by-products, and implement appropriate wastewater treatment strategies to ensure the safe disposal of treated effluents. Additionally, proper monitoring and regulation of g-C_3_N_4_-based photocatalytic processes are essential to prevent potential adverse impacts on human health and the environment.

Various experimental data show that g-C_3_N_4_-based materials are biocompatible only under the investigated doses. Biodegradability, chronic toxicity, and larger animal model testing have not been systematically and deeply studied yet. Therefore, the biocompatibility of g-C_3_N_4_-based photocatalysts still requires detailed biosafety assessments. Only when the biocompatibility of g-C_3_N_4_-based photocatalysts is fully demonstrated will it be possible to proceed with subsequent clinical applications [75]. For example, nanosheets of g-C_3_N_4_ exhibit promising applications as efficient photosensitizers for photodynamic tumour therapy and as pH-responsive nanocarriers for drug delivery. As photosensitizers, these nanosheets demonstrate the capability to generate ROS, effectively targeting and eliminating cancer cells under low-intensity light irradiation. Despite this mechanism holding significant potential for enhancing cancer treatment outcomes, the adverse effects on surrounding healthy tissues and cells need to be considered. However, when this material undergoes unintended processes such as the spontaneous release of certain components (e.g., metals) and the generation of ROS, its toxicity can be deemed a negative outcome [76].

Table 5 summarises possible mechanisms through which g-C_3_N_4_ could exhibit toxicity during biomedical applications (i.e., cancer therapy).

## 5. Assessment of g-C_3_N_4_ Toxicity

As mentioned above, in research for future practical use, the more attractive form of g-C_3_N_4_ has become the g-C_3_N_4_ nanosheets due to its improved properties such as higher photocatalytic activity or enhanced photoluminescence compared to bulk g-C_3_N_4_ material. Until now, g-C_3_N_4_ has been considered a nontoxic material, according to the following studies.

### 5.1. In Vitro Cytotoxicity Assessment on Human Cells

Thin nanosheets of g-C_3_N_4_ are considered a promising candidate within the field of bioimaging based on their various favourable attributes including enhanced photoluminescence, metal-free nature, and relatively low-cost preparation. An important step in researching the practical use of materials in bioimaging involves assessing cell toxicity. A summary of biocompatibility tests for g-C_3_N_4_ can be found in Table 6. Zhang et al. reported that ultrathin g-C_3_N_4_ nanosheets, prepared by ultrasound-assisted liquid exfoliation of bulk g-C_3_N_4_ in water, showed no evident reduction of HeLa cell viability even at a high incubation concentration of 600 μg/mL. The size distribution of tested exfoliated g-C_3_N_4_ was assessed by atomic force microscopy imaging, revealing a range from 70 to 160 nm, centred around 100 nm, and a thickness of nanosheets around 2.5 nm. The biocompatibility of prepared ultrathin nanosheets was further validated through a bioimaging test using an excitation wavelength *λ* = 405 nm, while the activity and morphology of HeLa cells were not disrupted [77].

A comparison of bulk material toxicity with the toxicity of nanosheets was tested on A549 cells, a lung epithelial carcinoma cell line, by Duan et al. The thickness of g-C_3_N_4_ nanosheets with a porous structure prepared by thermal exfoliation at 500 °C was determined as 1.0 nm. As the concentration of tested photocatalyst increased from 5 up to 100 μg/mL, the percentage of cell viability decreased after a 24-h incubation period irrespective of whether bulk or nanosheet material was used. Interestingly, g-C_3_N_4_ nanosheets were less destructive to A549 cells as the decrease in cell viability with increasing concentration was lower than bulk material. Even at the highest tested concentration of g-C_3_N_4_ nanosheets, the decrease in the A549 cell viability after 24 h was relatively low, which suggests good biocompatibility of g-C_3_N_4_ nanosheets toward tested cells within the tests [78].

Another study investigating the toxicity of bulk and g-C_3_N_4_ nanosheets was conducted by Davardoostmanesh et al. Nanosheets of a size under 100 nm were prepared by electrophoresis fractionation of bulk g-C_3_N_4_ synthesised from urea. The cytotoxicity tests of prepared materials were performed on Saos-2, a bone carcinoma cell line, and foreskin fibroblast HFF cells. Bulk g-C_3_N_4_ exhibited lower toxicity against Saos-2 cells after 24 and 48 h of incubation compared to g-C_3_N_4_ nanosheets. Determined half maximal inhibitory concentrations (IC_50_) were 66.8 ± 3.3 μg/mL and 27.0 ± 4.2 μg/mL for nanosheets and 214.4 ± 13.7 μg/mL and 104.0 ± 8.5 μg/mL for bulk after 24 and 48 h of incubation. Fluorescence microscopy revealed that g-C_3_N_4_ nanosheets were able to penetrate the tumour cells, distribute widely within the cytoplasm, and accumulate near the nuclear membrane. This finding was not observed with bulk material. There was no significant cell viability difference observed after treating normal HFF skin cells with 48-h IC_50_ concentrations of g-C_3_N_4_ in bulk or nanosheet form. Based on these discoveries, g-C_3_N_4_ nanosheets were proved to be potential candidates in bioimaging, drug delivery, and also anticancer therapy [79].

**Table 6:** Summary of g-C_3_N_4_ biocompatibility tests on human and animal cells.

Material Type	Cell Line	Methods	Concentration	Results	Ref.
g-C_3_N_4_ nanosheets	HeLa	MTT assay	600 μg/mL	No cytotoxic effect even at a high concentration of g-C_3_N_4_.	[77]
Bulk g-C_3_N_4_	A549	MTT assay	5–100 μg/mL	Dose-dependent decrease in cell viability.	[78]
g-C_3_N_4_ nanosheets				Better biocompatibility compared to bulk material.	
t-C_3_N_4_		MTT, WST-8 assay	25–500 μg/mL	t-C_3_N_4_ exhibited higher toxicity compared to h-C_3_N_4_.	[80]
h-C_3_N_4_					
Bulk g-C_3_N_4_	Saos-2	MTT assay, fluorescence microscopy	12.5–200 μg/mL	48-h IC_50_ = 104.0 ± 8.5 μg/mL.	[79]
g-C_3_N_4_ nanosheets				48-h IC_50_ = 27.0 ± 4.2 μg/mL.	
Bulk g-C_3_N_4_	HFF		48-h IC_50_	No cytotoxic effect.	
g-C_3_N_4_ nanosheets					
COOH-rich g-C_3_N_4_ nanosheets	MCF-7	CCK-8 assay, fluorescence microscopy	up to 400 μg/mL	No cytotoxic effect.	[81]
NH_3_-rich g-C_3_N_4_ nanosheets					
g-C_3_N_4_ nanosheets	HaCaT	MTS assay, optical microscopy	3.125–500 μg/mL	No cytotoxic effect.	[82]
Ni-doped g-C_3_N_4_ nanosheets				24-h IC_50_ = 53.93 μg/mL.	
Cu-doped g-C_3_N_4_ nanosheets				24-h IC_50_ = 157.00 μg/mL.	
Cu-Ni-doped g-C_3_N_4_ nanosheets				24-h IC_50_ = 40.10 μg/mL.	
g-C_3_N_4_ nanosheets	PC12	MTT assay	1–100 μg/mL	No significant cytotoxic effect under dark or white LED irradiation conditions.	[83]
Metal (Fe, Cu, Zn)-doped g-C_3_N_4_ nanosheets					
Cu-doped g-C_3_N_4_ nanosheets combined with upconversion nanoparticles	4T1	MTT assay	15.63–500 μg/mL	High cytotoxicity toward tumour 4T1 cells after NIR laser irradiation.	[84]
	L929			No obvious cytotoxic effect.	

In another study, Dong et al. examined and compared the cytotoxicity of g-C_3_N_4_ with triazine (t-C_3_N_4_) and heptazine (h-C_3_N_4_) structure moieties on the A549 cell line. According to the results obtained by MTT assay, t-C_3_N_4_ exhibits higher toxicity toward A549 cells compared to h-C_3_N_4_ across all tested concentrations ranging from 25 to 500 μg/mL. The same result was also achieved using the WST-8 assay after the same incubation time of 24 h as in the MTT assay. The suggested mechanism of t-C_3_N_4_ and h-C_3_N_4_ toxicity toward A549 cells via oxidative stress caused by ROS formation was confirmed by measurements of total glutathione in cells after incubation with both t-C_3_N_4_ and h-C_3_N_4_ at two different concentrations (50 and 100 μg/mL). It was proved that t-C_3_N_4_ induced higher levels of oxidative stress in A549 cells compared to h-C_3_N_4_, but both t-C_3_N_4_ and h-C_3_N_4_ showed significantly lower toxicity when compared to graphene oxide under similar experimental conditions [80].

Surface charge represents another important factor affecting the biological properties of g-C_3_N_4_. The relation between g-C_3_N_4_ surface charge and toxicity towards MCF-7 breast adenocarcinoma cells was investigated by Huang et al. Alteration in surface charge can be achieved through functionalisation via chemical oxidation to produce g-C_3_N_4_ rich in carboxyl groups (Cg-C_3_N_4_) or by chemical hydrolysis to create a nitrogen-rich derivate with amino groups (Ag-C_3_N_4_). Despite using the different synthesis methods, the obtained nanosheets were similar in size with 40–50 nm diameters. Neither of the prepared materials showed any toxicity effect and thus a significant reduction in cell viability after 24 h up to the concentration of 400 μg/mL. Cellular uptake examination by fluorescence spectroscopy and field emission scanning electron microscopy (FE-SEM) revealed that at equal concentrations, the Ag-C_3_N_4_ exhibited faster rates of cellular entry, which resulted in a higher accumulation of Ag-C_3_N_4_ within the cells. Additionally, at the lower incubation concentrations of g-C_3_N_4_, a larger contrast in cellular uptake was observed, suggesting that cationic Ag-C_3_N_4_ displays a greater affinity for cellular interactions because of the existing electrostatic interaction with the cell membrane charged negatively [81].

One of the other methods used for modifying the g-C_3_N_4_ structure is metal doping or codoping. The effect on the toxicity of g-C_3_N_4_ catalyst after metal nanoparticle doping was studied by Pieta et al. Cytotoxicity of prepared g-C_3_N_4_ and Ni-, Cu-, and Cu-Ni-doped g-C_3_N_4_ nanosheets, with sizes ranging from 1.1 to 1.5 nm, was investigated on the HaCaT human skin cell line. Microscopic analysis revealed that HaCaT cells incubated for 24 h with metal-doped g-C_3_N_4_ catalyst samples exhibited dose-dependent changes in morphology, while there was no evident change in morphology of cells observed after incubation with pure g-C_3_N_4_ nanosheets, even at the highest concentration of 500 μg/mL. In addition to the examination of HaCaT cell morphology, the impact of catalysts on the cell viability/proliferation was also determined using the MTS assay. A statistically significant reduction in cell viability was noted at concentrations of 25 μg/mL in the Ni-doped sample, 50 μg/mL in the Cu-doped sample, and 12.5 μg/mL in the Cu-Ni-codoped sample. In contrast, g-C_3_N_4_ nanosheets without modifications did not show any cytotoxic effect on HaCaT cells. The calculated IC_50_ values were 53.93 μg/mL for Ni-doped catalyst, 157.00 μg/mL for Cu-doped catalyst, and 40.10 μg/mL for Cu-Ni samples [82].

### 5.2. In Vitro Cytotoxicity Assessment on Animal Cells

In addition to the toxicity studies on human cell lines, a few studies have also examined the cytotoxic effects of g-C_3_N_4_ and its modifications on animal cells. Chung et al. studied the toxicity of g-C_3_N_4_ and metal (Fe, Cu, Zn)-doped g-C_3_N_4_ on rat pheochromocytoma PC12 cells. It was found that neither g-C_3_N_4_ nor its metal derivates caused a notable decrease in cell viability across the broad range of tested concentrations (0–100 μg/mL) under dark or under white LED irradiation conditions. Achieved results confirm good biocompatibility of g-C_3_N_4_ and metal-doped g-C_3_N_4_, as well as the negligible impact of ROS generated after irradiation of photocatalysts on animal cells [83].

The cytotoxicity of pyrrolic nitrogen-rich Cu-doped g-C_3_N_4_ nanosheets, in conjunction with upconversion nanoparticles (UCNPSs) acting as artificial enzymes, was tested on mouse cancerogenic 4 T1 and healthy L929 fibroblast cells. No apparent toxic effects of the prepared enzyme-like nanocomposite were observed in L929 cells after 24 h of incubation across a wide range of concentrations (0–500 μg/mL). Due to its good biocompatibility and ability to be taken up by 4 T1 cells, the nanocomposite with g-C_3_N_4_ was further investigated as a promising photosensitive drug against cancerous 4 T1 cells. Following irradiation with a 980 nm laser at varying concentrations of g-C_3_N_4_ nanocomposite, the 4 T1 cells were eliminated, while NIR laser irradiation alone resulted in a high survival of cells, indicating that 980 nm laser irradiation alone was ineffective in killing the cells [84].

### 5.3. Ecotoxicity Tests on Microorganisms, Fishes, and Plant Seeds

Besides the tests evaluating toxicity on various types of cells, it is crucial to investigate the ecotoxicity of g-C_3_N_4_ on various organisms, especially considering that a significant amount of g-C_3_N_4_ application research is focused on water treatment and environmental remediation. Rosa et al. evaluated the acute toxicity of g-C_3_N_4_ and magnetic nanocomposite consisting of g-C_3_N_4_ and Fe_3_O_4_ nanoparticles on zebrafish (*Danio rerio*) embryos. Bulk g-C_3_N_4_ used in the study was prepared by calcination of the mixture of melamine and urea, and the magnetic nanocomposites were prepared by the coprecipitation method. In the case of prepared samples of g-C_3_N_4_ at all tested concentrations (0–100 mg/L), no statistically significant changes were observed in all monitored parameters indicating lethality, developmental variations, or malformations during the development of the zebrafish embryo–larva 24, 48, 72, and 96 h after fertilisation. The results indicate that both g-C_3_N_4_ alone and in combination with magnetic nanoparticles do not cause teratogenic or acute toxic effects on the embryos of zebrafish [85].

The acute toxicity of ternary nanocomposite consisting of ZnO, CdO, and g-C_3_N_4_ was evaluated in Nile tilapia (*Oreochromis niloticus*) by Berhanu et al. Based on the observed results, g-C_3_N_4_ in combination with ZnO and CdO semiconductors does not cause any change in experimental toxicological parameters at low exposure concentrations (10 mg/L). However, at the higher concentrations (500 and 1000 mg/L) a few hours after exposure, tested fish started to show signs of respiratory distress such as dyspnoea, swimming to the water surface, or rapid movement of the operculum. The determined 50% lethal concentration (LC_50_) was at 12 h 113 mg/L [86].

Abdel-Moniem et al. analysed the toxicity of g-C_3_N_4_ and a nanocomposite consisting of Bi_2_S_3_ and g-C_3_N_4_ nanosheets using the Microtox model 500 analyser, capable of conducting in vitro tests of acute toxicity on the nonpathogenic bacterium *Vibrio fisheri*. The calculated effective 50% concentrations (EC_50_-15 min) of g-C_3_N_4_ nanosheets and Bi_2_S_3_-x@C_3_N_4_ nanosheets were ≥100, which indicates that these materials are non-toxic, environmentally friendly, and suitable for water treatment processes. Prepared samples were also tested on various microorganisms, specifically *Escherichia coli*, *Staphylococcus aureus*, and *Candida albicans*, for disinfecting properties under dark conditions. Out of all tested materials, g-C_3_N_4_ nanosheets displayed the weakest antimicrobial properties against all selected microorganisms. Its higher antimicrobial activity was observed against *E. coli*, with the value almost reaching 80%. However, the antimicrobial activity against *S. aureus* and *C. albicans* was negligible, not even reaching 20%. Meanwhile, the antimicrobial activity of the g-C_3_N_4_ nanosheets modified with Bi_2_S_3_ nanosheets significantly improved, with the values almost reaching up to 100% toward all tested microorganisms [87].

The possible phytotoxicity of g-C_3_N_4_ toward rice seedlings (*Oryza sativa* L.) was evaluated by Hao et al. Tested g-C_3_N_4_, with an average size spanning several hundred nanometres and thickness of about 3–7 nm, was synthesised by urea calcination followed by freeze-drying. The obtained weights of rice biomass, treated with g-C_3_N_4_ (250 mg/kg), after 30 days of exposure showed increases of 29.9% (rice roots), and 22.4% (rice shoots) compared to untreated control rice seedlings. The utilisation of a low dose of g-C_3_N_4_ did not affect the rice seedlings’ growth and it even mitigated the phytotoxicity induced by heavy metals (As, Cd) present in the soil through adsorption of metals on g-C_3_N_4_, which caused inhibition of As and Cd distribution and accumulation in rice plants [88].

## 6. Conclusions and Future Perspectives

**Figure 4:**
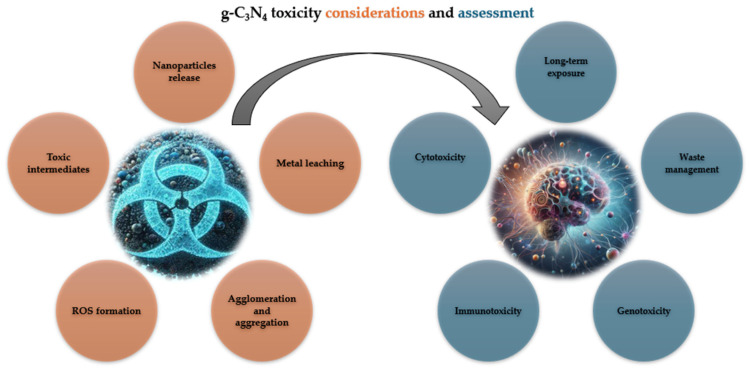
Schematic illustration of g-C_3_N_4_ potential toxicity routes and evaluation methods.

The prospects of g-C_3_N_4_ lie in its further development and improvement for a variety of applications such as photocatalysis, water splitting, environmental remediation, and even sensor applications. The sensitivity of g-C_3_N_4_ towards various analytes, including organic compounds and heavy metals, has been demonstrated, making g-C_3_N_4_ a suitable material useful in processes such as environmental monitoring, healthcare diagnostics, and food safety testing. Overall, while g-C_3_N_4_ is considered to be relatively safe for many applications, it is also essential to carefully evaluate the potential toxicity risks associated with its use (Figure 4) in sensing and electronics applications. The potential toxicity of these nanocomposites may depend on factors such as the size, shape, surface chemistry, and dose of the g-C_3_N_4_ nanoparticles, as well as the specific application and exposure scenario. Understanding whether g-C_3_N_4_-containing materials can be recycled or safely disposed of at the end of their lifecycle is important for sustainable product stewardship. Proper waste management practices should be followed to minimise the release of g-C_3_N_4_ nanoparticles into the environment and mitigate potential ecological risks. In biomedical applications, such as drug delivery systems or biomedical imaging, the potential toxicity of g-C_3_N_4_ nanoparticles to human cells or tissues needs to be thoroughly evaluated. This includes assessing cytotoxicity, genotoxicity, immunotoxicity, and any potential long-term health effects associated with exposure to g-C_3_N_4_ materials.

g-C_3_N_4_ is often used in photocatalytic coatings for environmental remediation purposes, such as air purification or water treatment. While g-C_3_N_4_ itself is considered to be relatively inert, there may be concerns about the release of nanoparticles, metals, or degradation products from these coatings into the environment, which could potentially impact human health or ecosystems. Once g-C_3_N_4_ is doped with heavy metals such as cadmium to enhance its photocatalytic activity for water purification, there is a risk of leaching of toxic metal ions into the environment during the catalytic process. These metal ions can pose significant environmental and health hazards due to their toxicity and potential for bioaccumulation in organisms. If released into water bodies, the metal ions could contaminate the aquatic environment, posing risks to aquatic organisms and potentially entering the food chain.

Understanding the mechanical properties of g-C_3_N_4_ is essential for ensuring the robustness, proper design, and optimal functioning of coatings, devices, and systems based on this material. The mechanical characterisation of g-C_3_N_4_ reveals a material with high stiffness, moderate hardness, and good wear resistance, suitable for a range of applications from coatings to structural components. However, its brittle nature and anisotropic properties need careful consideration in design and application. Researchers and engineers can optimise g-C_3_N_4_ for specific uses by understanding and leveraging these mechanical properties, ensuring robustness and longevity in practical applications. Exploring modifications, heterojunctions, novel composite structures, and nanostructures for enhancing g-C_3_N_4_ efficiency will allow the expansion of its practical applications in sustainable energy and environmental management. Overall, the perspectives of g-C_3_N_4_ are vast and multidisciplinary. Continued research efforts aimed at the examination of its properties and synthesis methods as well as toxicity investigations are necessary for further progress.

## Figures and Tables

**Table 1:** Synthesis methodologies of g-C_3_N_4_ and their outcomes [5,6,25,27].

Synthesis Type	Result	Efficiency and Purity
Thermal polymerisation	Produces bulk g-C_3_N_4_ with high crystallinity and decent photocatalytic properties. However, this method often results in a limited surface area and poor dispersion in solutions.	High purity with moderate efficiency due to limited surface area and electron–hole recombination issues.
Solvothermal and hydrothermal methods	Creates g-C_3_N_4_ with various morphologies, such as nanosheets or nanospheres, and can introduce porosity.	Higher surface area and enhanced photocatalytic activity compared to bulk g-C_3_N_4_. Purity depends on the solvent and reaction conditions but is generally good.
Template-assisted synthesis	Produces porous or structured g-C_3_N_4_ with high surface areas and tailored morphologies.	High surface area leads to improved catalytic performance. Template removal can impact purity if not performed thoroughly.
Chemical vapour deposition (CVD)	Yields thin films of g-C_3_N_4_ with controlled thickness and high uniformity.	Very high purity and good control over material properties, leading to efficient catalytic and electronic applications.
Molten salt synthesis	Produces g-C_3_N_4_ with a high degree of crystallinity and large surface area.	High efficiency due to better dispersion and surface properties. Purity is generally high but depends on the post-synthesis washing to remove residual salts.
Doping and composite formation	Enhances electronic properties, photocatalytic activity, and stability. Creates composites with synergistic properties.	Improved efficiency due to enhanced charge separation and increased active sites. Purity can be impacted by the choice of dopants and composite materials.
Exfoliation techniques	Produces ultrathin g-C_3_N_4_ nanosheets with high surface areas and excellent catalytic properties.	High efficiency due to increased active sites and better light absorption. Purity is generally high if exfoliation is performed without introducing impurities.

**Table 2:** Sensing applications of g-C_3_N_4_ (mentioned in Section 3.1 and Section 3.2) and their performance comparison to other photocatalytic materials.

Analyte	g-C_3_N_4_-Based Sensing Platform	Ref.	LOD (μM)	Comparison Material	LOD (μM)	Ref.
Tryptophan	g-C_3_N_4_-modified CPE	[29]	0.085	Graphene-modified CGE	0.3	[44]
Acyclovir	g-C_3_N_4_-modified CPE	[30]	3 × 10^−3^	Magnetic CdO NPs-modified GCE	0.3	[45]
Heavy metals (Cr, Ni)	g-C_3_N_4_, MWCNT-modified CPE	[31]	6.7 × 10^−3^ and 0.012	Zeolite and chlorinated MWCNT-modified CPE	0.06 (Cr)	[46]
Dibenzofuran	Oxygenated g-C_3_N_4_-modified SPE	[32]	1.58 × 10^−6^	Silver electrode modified with MnO_2_ nanofibers	1.2 × 10^−3^	[47]
2,4-dichlorophenol	Fe-MOF/g-C_3_N_4_-modified SPE	[33]	1.2 × 10^−3^	Cu-MOF/rGO composite-modified GCE	0.083	[48]
Iodide ions (I^−^)	g-C_3_N_4_/chitosan-modified SPE	[34]	0.01	Silver oxide microparticles PAA/PVA-modified GCE	0.3	[49]
Metronidazole	MoS_2_/g-C_3_N_4_-modified GCE	[35]	0.09	Graphene nanosheets/Fe_3_O_4_ modified-GCE	0.23 × 10^−3^	[50]
Epinephrine	Bi_2_Te_3_ /g-C_3_N_4_-modified GCE	[36]	0.71	Zeolite imidazole framework on GCE	2.1	[51]
Perphenazine	LaCoO_3_/g-C_3_N_4_-modified GCE	[37]	4.3 × 10^−3^	Graphene oxide nanosheets on GCE	38.4	[52]
Ascorbic acid	FTO glass/g-C_3_N_4_/BiOI PEC platform	[40]	3.3	Cu-porphyrin MOF	0.023	[53]
P-nitroroluene	g-C_3_N_4_ nanosheets/Cu(2%) PEC platform	[41]	0.13	-	-	-
Atrazine	g-C_3_N_4_ nanosheets doped with cyano groups and N deficiencies on ITO glass PEC aptasensor	[42]	3.33 × 10^−11^	BiOI nanoflowers/TiO_2_ nanotubes PEC platform	0.5 × 10^−6^	[54]
Aflatoxin B1	g-C_3_N_4_ + aptamer on GCE	[43]	0.005 ng/mL	Methylamine perovskite quantum dots, encapsulated by ZIF-8 MOF on GCE	3.5 fg/mL	[55]

**Table 3:** Properties of common water-splitting catalysts compared to g-C_3_N_4_ [68,69,70,71].

Material Type	Bandgap (eV)	Stability	Cost and Scalability	Environmental Impact	Other Drawbacks
g-C_3_N_4_	2.7—easily excited by visible light.	Thermally and chemically stable.	Cheap and abundant precursors, relatively simple synthesis, effective for large-scale applications.	Environmentally compatible.	Prone to rapid electron–hole recombination, functionalisation is necessary to overcome drawbacks.
Metal oxides	Several catalysts such as TiO_2,_ SnO_2,_ ZnO, and NiO are limited by larger bandgaps (>3.2)—not able to adsorb visible light.	Extremely stable.	Limited availability and high cost of several noble metals (Pt, Ru, Ir, Pd). Difficult for large-scale production.	Can be environmentally hazardous due to the potential release of toxic heavy metals.	Prone to deactivation due to adsorption of impurities or changes in pH. In nanoparticle form, prone to agglomeration.
Metal chalcogenides	Typically a much narrower bandgap (1.2–2.4).	Susceptible to photocorrosion and environmental degradation.	Several metals are expensive and low in abundance. Difficult for large-scale production.	Can be environmentally hazardous due to the potential release of toxic heavy metals.	Limited activity, low conductivity, low synthesis yield.
Perovskite materials	Adjustable, depending on the composition.	Prone to degradation from oxygen, moisture, UV exposure, and high temperatures (above 80).	Low cost of materials, faces challenges in large-scale processing.	Some perovskites contain lead. Additionally, toxic solvents are employed in processing.	Low catalytic performance when used alone.
Porous organic polymers (POPs)	Adjustable, depending on composition.	Generally stable.	Inexpensive precursors, scalable. Estimated production cost lower than 10 USD/kg.	Generally nontoxic, energy-efficient, and recyclable, though case-specific.	Relatively large pore size (>1 nm), which may be a limiting factor in some applications.

**Table 4:** g-C_3_N_4_ mechanisms and toxicity considerations during photocatalytic processes.

Risk	Impact
Toxic intermediates	As phenol undergoes degradation, various intermediate products are formed. Some of these intermediates may exhibit toxicity to humans and aquatic organisms, potentially posing risks to environmental and human health.
Incomplete degradation	Incomplete degradation of phenol or its intermediates could result in the accumulation of persistent organic pollutants in the environment, leading to long-term ecological impacts and potential bioaccumulation in the food chain.
Unwanted ROS	Uncontrolled generation of ROS during the photocatalytic process may cause oxidative stress in aquatic organisms and disrupt ecosystems, particularly in sensitive aquatic environments.
Release of nanoparticles	While g-C_3_N_4_ is generally considered to be biocompatible, the long-term effects of nanoparticle exposure on human health and the environment are still not fully understood.

**Table 5:** g-C_3_N_4_ mechanisms and toxicity considerations in biomedical applications.

Consideration	Impact
Particle size and shape	Nanoparticles of g-C_3_N_4_ may exhibit different properties and behaviours compared to bulk materials. Their small size and high surface area-to-volume ratio could increase interactions with biological systems, potentially leading to adverse effects such as cellular uptake, oxidative stress, and inflammation.
Chemical composition	The chemical composition of g-C_3_N_4_, including any surface functionalisation or impurities, could influence its toxicity profile. For example, surface groups or contaminants may enhance cellular uptake or trigger immune responses, leading to cytotoxic or immunotoxic effects.
Biological interactions	When introduced into biological systems, g-C_3_N_4_ nanoparticles may interact with cellular components such as proteins, lipids, and nucleic acids. These interactions could disrupt cellular processes, interfere with signalling pathways, or induce cellular damage, ultimately leading to cytotoxicity or genotoxicity.
Oxidative stress	Nanoparticles of g-C_3_N_4_ have the potential to generate ROS through photoactivation or chemical reactions. Excessive ROS production can overwhelm cellular antioxidant defences, leading to oxidative stress and cellular damage.
Biodegradation and clearance	The biodegradation and clearance of g-C_3_N_4_ nanoparticles from the body are critical factors in determining their long-term toxicity. If nanoparticles persist in biological tissues or accumulate in organs over time, they may elicit adverse effects such as chronic inflammation, fibrosis, or organ damage.
Aggregation and agglomeration	Nanoparticles of g-C_3_N_4_ may agglomerate or aggregate in biological fluids or tissues, altering their physicochemical properties and biological interactions. Aggregated nanoparticles could lead to localised toxicity, impaired cellular uptake, or obstruction of biological pathways.

## References

[1] Wang S., Wang L., Cong H., Wang R., Yang J., Li X., Zhao Y., Wang H. (2022). A review: G-C_3_N_4_ as a new membrane material. J. Environ. Chem. Eng..

[2] Nasir M.S., Yang G., Ayub I., Wang S., Wang L., Wang X., Yan W., Peng S., Ramakarishna S. (2019). Recent development in graphitic carbon nitride based photocatalysis for hydrogen generation. Appl. Catal. B Environ..

[3] Wang N., Cheng L., Liao Y., Xiang Q. (2023). Effect of Functional Group Modifications on the Photocatalytic Performance of g-C_3_N_4_. Small.

[4] Miller T.S., Jorge A.B., Suter T.M., Sella A., Corà F., McMillan P.F. (2017). Carbon nitrides: Synthesis and characterization of a new class of functional materials. Phys. Chem. Chem. Phys..

[5] Bhanderi D., Lakhani P., Modi C.K. (2024). Graphitic carbon nitride (g-C_3_N_4_) as an emerging photocatalyst for sustainable environmental applications: A comprehensive review. RSC Sustain..

[6] Zhu J., Xiao P., Li H., Carabineiro S.A.C. (2014). Graphitic carbon nitride: Synthesis, properties, and applications in catalysis. ACS Appl. Mater. Interfaces.

[7] Luo Y., Yan Y., Zheng S., Xue H., Pang H. (2019). Graphitic carbon nitride based materials for electrochemical energy storage. J. Mater. Chem. A.

[8] Liu H., Wang X., Wang H., Nie R. (2019). Synthesis and biomedical applications of graphitic carbon nitride quantum dots. J. Mater. Chem. B.

[9] Ong W.J., Tan L.L., Ng Y.H., Yong S.T., Chai S.P. (2016). Graphitic carbon nitride (g-C_3_N_4_)-based photocatalysts for artificial photosynthesis and environmental remediation: Are we a step closer to achieving sustainability?. Chem. Rev..

[10] Fang L., Ohfuji H., Shinmei T., Irifune T. (2011). Experimental study on the stability of graphitic C_3_N_4_ under high pressure and high temperature. Diam. Relat. Mater..

[11] Zheng Y., Zhang Z., Li C. (2017). A comparison of graphitic carbon nitrides synthesized from different precursors through pyrolysis. J. Photochem. Photobiol. A Chem..

[12] Sharma P., Sarngan P.P., Lakshmanan A., Sarkar D. (2022). One-step synthesis of highly reactive gC_3_N_4_. J. Mater. Sci. Mater. Electron..

[13] Hu C., Chu Y.C., Wang M.S., Wu X.H. (2017). Rapid synthesis of g-C_3_N_4_ spheres using microwave-assisted solvothermal method for enhanced photocatalytic activity. J. Photochem. Photobiol. A Chem..

[14] Xu T., Hur J., Niu P., Wang S., Lee S., Chun S.E., Li L. (2024). Synthesis of crystalline g-C_3_N_4_ with rock/molten salts for efficient photocatalysis and piezocatalysis. Green Energy Environ..

[15] Yadav R.M., Kumar R., Aliyan A., Dobal P.S., Biradar S., Vajtai R., Singh D.P., Martí A.A., Ajayan P.M. (2020). Facile synthesis of highly fluorescent free-standing films comprising graphitic carbon nitride (g-C_3_N_4_) nanolayers. New J. Chem..

[16] Umapathi R., Raju C.V., Ghoreishian S.M., Rani G.M., Kumar K., Oh M.H., Park J.P., Huh Y.S. (2022). Recent advances in the use of graphitic carbon nitride-based composites for the electrochemical detection of hazardous contaminants. Coord. Chem. Rev..

[17] Pourmadadi M., Rahmani E., Eshaghi M.M., Shamsabadipour A., Ghotekar S., Rahdar A., Ferreira L.F.R. (2023). Graphitic carbon nitride (g-C_3_N_4_) synthesis methods, surface functionalization, and drug delivery applications: A review. J. Drug Deliv. Sci. Technol..

[18] Nihal, Sharma R., Kaur N., Sharma M., Choudhary B.C., Goswamy J.K. (2023). Transition metal (Ni, Pd and Pt)-embedded graphitic carbon nitride (gCN) monolayer as an acetone sensor: A computational and experimental study. J. Mater. Sci. Mater. Electron..

[19] Molaei M.J. (2023). Graphitic carbon nitride (g-C_3_N_4_) synthesis and heterostructures, principles, mechanisms, and recent advances: A critical review. Int. J. Hydrogen Energy.

[20] Niu P., Zhang L., Liu G., Cheng H.M. (2012). Graphene-like carbon nitride nanosheets for improved photocatalytic activities. Adv. Funct. Mater..

[21] Chebanenko M.I., Omarov S.O., Lobinsky A.A., Nevedomskiy V.N., Popkov V.I. (2023). Steam exfoliation of graphitic carbon nitride as efficient route toward metal-free electrode materials for hydrogen production. Int. J. Hydrogen Energy.

[22] Torres-Pinto A., Silva C.G., Faria J.L., Silva A.M. (2023). The effect of precursor selection on the microwave-assisted synthesis of graphitic carbon nitride. Catal. Today.

[23] Yang Z., Zhang Y., Schnepp Z. (2015). Soft and hard templating of graphitic carbon nitride. J. Mater. Chem. A Mater. Energy Sustain..

[24] Lu X., Xu K., Chen P., Jia K., Liu S., Wu C. (2014). Facile one step method realizing scalable production of g-C_3_N_4_ nanosheets and study of their photocatalytic H2evolution activity. J. Mater. Chem. A Mater. Energy Sustain..

[25] Chen L., Maigbay M.A., Li M., Qiu X. (2024). Synthesis and modification strategies of g-C3N4 nanosheets for photocatalytic applications. Adv. Powder Mater..

[26] Mo Z., Zhu X., Jiang Z., Song Y., Liu D., Li H., Yang X., She Y., Lei Y., Yuan S. (2019). Porous nitrogen-rich g-C_3_N_4_ nanotubes for efficient photocatalytic CO_2_ reduction. Appl. Catal. B.

[27] Chen Z., Zhang S., Liu Y., Alharbi N.S., Rabah S.O., Wang S., Wang X. (2020). Synthesis and fabrication of g-C_3_N_4_-based materials and their application in elimination of pollutants. Sci. Total Environ..

[28] Vinoth S., Devi K.S., Pandikumar A. (2021). A comprehensive review on graphitic carbon nitride-based electrochemical and biosensors for environmental and healthcare applications. Trends Analyt. Chem..

[29] Abebe H.A., Diro A., Kitte S.A. (2023). Voltammetric determination of tryptophan at graphitic carbon nitride modified carbon paste electrode. Heliyon.

[30] Sreenivasulu M., Malode S.J., Alqarni S.A., Shetti N.P. (2024). Graphitic carbon nitride (g–C_3_N_4_)-based electrochemical sensors for the determination of antiviral drug acyclovir. Mater. Chem. Phys..

[31] Sharif Manesh S., Masrournia M. (2021). Carbon nitride nanoparticles modified carbon paste electrodes as potentiometric sensors for determination of nickel (II) and chromium (III) ions in tap water samples. J. Iran. Chem. Soc..

[32] Singh S., Naithani A., Kandari K., Roy S., Sain S., Roy S.S., Wadhwa S., Tauseef S.M., Mathur A. (2023). Oxygenated graphitic carbon nitride based electro-chemical sensor for dibenzofuran detection. Diam. Relat. Mater..

[33] Ambaye A.D., Kebede T.G., Ntsendwana B., Nxumalo E.N. (2023). Fe-MOF derived graphitic carbon nitride nanocomposites as novel electrode materials for the electrochemical sensing of 2, 4-dichlorophenol in wastewater. Synth. Met..

[34] Niaz A., Arain M.B., Soylak M. (2024). Sensitive determination of iodide at graphitic carbon nitride-chitosan composite modified screen-printed electrode in urine and salt using cathodic stripping voltammetry. Microchem. J..

[35] Ahmad K., Raza W., Alsulmi A., Kim H. (2023). Fabrication of electrochemical sensor for metronidazole using MoS_2_/graphite-like carbon nitride composite modified glassy carbon electrode. Diam. Relat. Mater..

[36] Dasi A., Asadpour-Zeynali K., Saeb E. (2024). Preparation of a fast and simple electrochemical sensor of bismuth telluride decorated on graphitic carbon nitride nanosheets for determination of epinephrine in biological samples. Synth. Met..

[37] Koventhan C., Shanmugam R., Chen S.M. (2023). Development of highly sensitive electrochemical sensor for antipsychotic drug perphenazine using perovskite structured lanthanum cobalt oxide nanoparticles wrapped graphitic carbon nitride nanocomposites. Electrochim. Acta.

[38] Svitkova V., Palchetti I. (2020). Functional polymers in photoelectrochemical biosensing. Bioelectrochemistry.

[39] Svitková V., Konderíková K., Nemčeková K. (2022). Photoelectrochemical aptasensors for detection of viruses. Monatsh. Chem..

[40] Li W., Zhang M., Han D., Yang H., Hong Q., Fang Y., Zhou Z., Shen Y., Liu S., Huang C. (2023). Carbon nitride-based heterojunction photoelectrodes with modulable charge-transfer pathways toward selective biosensing. Anal. Chem..

[41] Chen L., Li Z., Xiao Q., Li M., Xu Y., Qiu X. (2023). Sensitive detection of p-nitrotoluene based on a copper cluster modified carbon nitride nanosheets photoelectrochemical sensor. Appl. Catal. A Gen..

[42] Yan P., Jin Y., Xu L., Mo Z., Qian J., Chen F., Yuan J., Xu H., Li H. (2022). Enhanced photoelectrochemical aptasensing triggered by nitrogen deficiency and cyano group simultaneously engineered 2D carbon nitride for sensitively monitoring atrazine. Biosens. Bioelectron..

[43] Tian D., Wang J., Zhuang Q., Wu S., Yu Y., Ding K. (2023). An electrochemiluminescence biosensor based on Graphitic carbon nitride luminescence quenching for detection of AFB1. Food Chem..

[44] Pogacean F., Varodi C., Coros M., Kacso I., Radu T., Cozar B.I., Mirel V., Pruneanu S. (2021). Investigation of L-tryptophan electrochemical oxidation with a graphene-modified electrode. Biosensors.

[45] Naghian E., Marzi Khosrowshahi E., Sohouli E., Pazoki-Toroudi H.R., Sobhani-Nasab A., Rahimi-Nasrabadi M., Ahmadi F. (2020). Electrochemical oxidation and determination of antiviral drug acyclovir by modified carbon paste electrode with magnetic CdO nanoparticles. Front. Chem..

[46] Heidari Z., Masrournia M. (2018). A novel modified carbon paste electrode for the determination of chromium(III) in water. J. Anal. Chem..

[47] Gupta A.K., Roy S., Nagabooshanam S., Wadhwa S., Aravindan S., Singh D., Mathur A., Kumar R. (2020). Label-Free Electrochemical Detection of Dibenzofuran Using MnO_2_ Nanofibres. IEEE Sens. J..

[48] Nguyen M.B., Hong Nhung V.T., Thu V.T., Ngoc Nga D.T., Pham Truong T.N., Giang H.T., Hai Yen P.T., Phong P.H., Vu T.A., Thu Ha V.T. (2020). An electrochemical sensor based on copper-based metal–organic framework-reduced graphene oxide composites for determination of 2,4-dichlorophenol in water. RSC Adv..

[49] Khunseeraksa V., Kongkaew S., Thavarungkul P., Kanatharana P., Limbut W. (2020). Electrochemical sensor for the quantification of iodide in urine of pregnant women. Mikrochim. Acta.

[50] Zokhtareh R., Rahimnejad M., Najafpour-Darzi G., Karimi-Maleh H. (2023). A novel sensing platform for electrochemical detection of metronidazole antibiotic based on green-synthesized magnetic Fe_3_O_4_ nanoparticles. Environ. Res..

[51] Soosaimanickam C., Sakthivel A., Murugavel K., Alwarappan S. (2023). Zeolite imidazolate framework-based platform for the electrochemical detection of epinephrine. J. Electrochem. Soc..

[52] Heli H., Sattarahmady N., Zare S.N. (2015). Electrooxidation and determination of perphenazine on a graphene oxide nanosheet-modified electrode. RSC Adv..

[53] Xu X., Li C.H., Zhang H., Guo X.M. (2022). Construction of electrochemical and photoelectrochemical sensing platform based on porphyrinic metal-organic frameworks for determination of ascorbic acid. Nanomaterials.

[54] Fan L., Liang G., Zhang C., Fan L., Yan W., Guo Y., Shuang S., Bi Y., Li F., Dong C. (2021). Visible-light-driven photoelectrochemical sensing platform based on BiOI nanoflowers/TiO2 nanotubes for detection of atrazine in environmental samples. J. Hazard. Mater..

[55] Wang Q., Xiong C., Li J., Deng Q., Zhang X., Wang S., Chen M.M. (2023). High-performance electrochemiluminescence sensors based on ultra-stable perovskite quantum dots@ZIF-8 composites for aflatoxin B1 monitoring in corn samples. Food Chem..

[56] Cheng L., Zhang H., Li X., Fan J., Xiang Q. (2021). Carbon–graphitic carbon nitride hybrids for heterogeneous photocatalysis. Small.

[57] Tang C., Cheng M., Lai C., Li L., Yang X., Du L., Zhang G., Wang G., Yang L. (2023). Recent progress in the applications of non-metal modified graphitic carbon nitride in photocatalysis. Coord. Chem. Rev..

[58] Xu Q., Dai L., Wang Z., Wu J., Lu H., Yuan L., Zhu Q., Zeng X. (2023). Renewable ultrathin carbon nitride nanosheets and its practical utilization for photocatalytic decarboxylation free radical coupling reaction. Chem. Eng. J..

[59] Yuan X., Xie R., Zhang Q., Sun L., Long X., Xia D. (2019). Oxygen functionalized graphitic carbon nitride as an efficient metal-free ozonation catalyst for atrazine removal: Performance and mechanism. Sep. Purif. Technol..

[60] Fernandes E., Mazierski P., Miodyńska M., Klimczuk T., Zaleska-Medynska A., Oliveira J., Matos A.M., Martins R.C., Gomes J. (2024). Emerging contaminants and pathogenic microorganisms elimination in secondary effluent by graphitic carbon nitride photocatalytic ozonation processes. Catal. Today.

[61] Xu Q., Wu J., Qian Y., Chen X., Han Y., Zeng X., Qiu B., Zhu Q. (2024). Order-Disorder Engineering of Carbon Nitride for Photocatalytic H2O2 Generation Coupled with Pollutant Removal. ACS Appl. Mater. Interfaces.

[62] Zhang C., Li Y., Shuai D., Shen Y., Xiong W., Wang L. (2019). Graphitic carbon nitride (g-C_3_N_4_)-based photocatalysts for water disinfection and microbial control: A review. Chemosphere.

[63] Hota P., Das A., Maiti D.K. (2023). A short review on generation of green fuel hydrogen through water splitting. Int. J. Hydrogen Energy.

[64] Mohan A.A., Sandhyarani N. (2023). Carbon nanostructures for energy generation and storage. Applications of Multifunctional Nanomaterials.

[65] Wang T.H., Nguyen T.K.A., Doong R.A. (2022). Phosphorene nanosheet decorated graphitic carbon nitride nanofiber for photoelectrochemically enhanced hydrogen evolution from water splitting. J. Taiwan Inst. Chem. Eng..

[66] Sun D., Chen Y., Yu X., Yin Y., Tian G. (2023). Engineering high-coordinated cerium single-atom sites on carbon nitride nanosheets for efficient photocatalytic amine oxidation and water splitting into hydrogen. Chem. Eng. J..

[67] Torres-Pinto A., Díez A.M., Silva C.G., Faria J.L., Sanromán M.Á., Silva A.M., Pazos M. (2024). Tuning graphitic carbon nitride (g-C_3_N_4_) electrocatalysts for efficient oxygen evolution reaction (OER). Fuel.

[68] Wang S., Lu A., Zhong C.J. (2021). Hydrogen production from water electrolysis: Role of catalysts. Nano Converg..

[69] Yin J., Jin J., Lin H., Yin Z., Li J., Lu M., Guo L., Xi P., Tang Y., Yan C.H. (2020). Optimized metal chalcogenides for boosting water splitting. Adv. Sci..

[70] Huang Y., Liu J., Deng Y., Qian Y., Jia X., Ma M., Yang C., Liu K., Wang Z., Qu S. (2020). The application of perovskite materials in solar water splitting. J. Semicond..

[71] Luo D., Shi T., Li Q.H., Xu Q., Strømme M., Zhang Q.F., Xu C. (2023). Green, general and low-cost synthesis of porous organic polymers in sub-kilogram scale for catalysis and CO_2_ capture. Angew. Chem. Int. Ed..

[72] Perveen M., Nazir S., Arshad A.W., Khan M.I., Shamim M., Ayub K., Khan M.A., Igbal J. (2020). Therapeutic potential of graphitic carbon nitride as a drug delivery system for cisplatin (anticancer drug): A DFT approach. Biophys. Chem..

[73] Cheng H.-L., Guo H.-L., Xie A.-J., Shen Y.-H., Zhu M.-Z. (2021). 4-in-1 Fe_3_O_4_/g-C_3_N_4_@PPy-DOX nanocomposites: Magnetic targeting guided trimode combinatorial chemotherapy/PDT/PTT for cancer. J. Inorg. Biochem..

[74] Ali I., Kim J.-O. (2021). Optimization of photocatalytic performance of a gC_3_N_4_–TiO_2_ nanocomposite for phenol degradation in visible light. Mat. Chem. Phys..

[75] Che S., Zhang L., Wang T., Su D., Wang C. (2021). Graphitic Carbon Nitride-Based Photocatalysts for Biological Applications. Adv. Sustain. Syst..

[76] Lin L.-S., Cong Z.-X., Li J., Ke K.-M., Guo S.-S., Yang H.-H., Chen G.-N. (2014). Graphitic-phase C_3_N_4_ nanosheets as efficient photosensitizers and pH-responsive drug nanocarriers for cancer imaging and therapy. J. Mater. Chem. B.

[77] Zhang X., Xie X., Wang H., Zhang J., Pan B., Xie Y. (2013). Enhanced photoresponsive ultrathin graphitic-phase C_3_N_4_ nanosheets for bioimaging. J. Am. Chem. Soc..

[78] Duan Y., Zhou S., Deng L., Shi Z., Jiang H., Zhou S. (2020). Enhanced photocatalytic degradation of sulfadiazine via g-C_3_N_4_/carbon dots nanosheets under nanoconfinement: Synthesis, Biocompatibility and Mechanism. J. Environ. Chem. Eng..

[79] Davardoostmanesh M., Ahmadzadeh H., Goharshadi E.K., Meshkini A., Sistanipour E. (2020). Graphitic carbon nitride nanosheets prepared by electrophoretic size fractionation as an anticancer agent against human bone carcinoma. Mater. Sci. Eng. C Mater. Biol. Appl..

[80] Dong Q., Latiff N.M., Mazánek V., Rosli F.R., Chia H.L., Sofer Z., Pumera M. (2018). Triazine- and heptazine-based carbon nitrides: Toxicity. ACS Appl. Nano Mater..

[81] Huang Q., Hao L., Zhou R., Zhu B., Zhao H., Cai X. (2018). Synthesis, characterization, and biological study of carboxyl- and amino-rich g-C_3_N_4_ nanosheets by different processing routes. J. Biomed. Nanotechnol..

[82] Pieta I.S., Gieroba B., Kalisz G., Pieta P., Nowakowski R., Naushad M., Rathi A., Gawande M.B., Sroka-Bartnicka A., Zboril R. (2022). Developing benign Ni/g-C_3_N_4_ catalysts for CO_2_ hydrogenation: Activity and toxicity study. Ind. Eng. Chem. Res..

[83] Chung Y.J., Lee B.I., Ko J.W., Park C.B. (2016). Photoactive g-C_3_N_4_ nanosheets for light-induced suppression of Alzheimer’s β-amyloid aggregation and toxicity. Adv. Healthc. Mater..

[84] Song S., Yang M., He F., Zhang X., Gao Y., An B., Ding H., Gai S., Yang P. (2023). Multiple therapeutic mechanisms of pyrrolic N-rich g-C_3_N_4_ nanosheets with enzyme-like function in the tumor microenvironment. J. Colloid Interface Sci..

[85] Rosa E.V., Fascineli M.L., Silva I.C.R., Rodrigues M.O., Chaker J.A., Grisolia C.K., Moya S.E., Campos A.F.C., Sousa M.H. (2021). Carbon nitride nanosheets magnetically decorated with Fe_3_O_4_ nanoparticles by homogeneous precipitation: Adsorption-photocatalytic performance and acute toxicity assessment. Environ. Nanotechnol. Monit. Manag..

[86] Berhanu S., Gebremariam H., Chufamo S. (2022). The g-C_3_N_4_@CdO/ZnO ternary composite: Photocatalysis, thermodynamics and acute toxicity studies. Heliyon.

[87] Abdel-Moniem S.M., El-Liethy M.A., Ibrahim H.S., Ali M.E.M. (2021). Innovative green/non-toxic Bi_2_S_3_@g-C_3_N_4_ nanosheets for dark antimicrobial activity and photocatalytic depollution: Turnover assessment. Ecotoxicol. Environ. Saf..

[88] Hao Y., Cai Z., Ma C., White J.C., Cao Y., Chang Z., Xu X., Han L., Jia W., Zhao J. (2023). Root exposure of graphitic carbon nitride (g-C_3_N_4_) modulates metabolite profile and endophytic bacterial community to alleviate cadmium- and arsenate-induced phytotoxicity to rice (*Oryza sativa* L.). ACS Nano.

